# Development of a hospital frailty risk score for community-dwelling older adults using data from electronic hospital records in South Korea

**DOI:** 10.1371/journal.pone.0293646

**Published:** 2023-11-02

**Authors:** Hee-Sun Kim, Jinhee Kim, Gihwan Bae

**Affiliations:** 1 National Evidence-Based Collaborating Agency, Seoul, South Korea; 2 Department of Nursing, Chosun University Hospital, Gwangju, South Korea; 3 Artificial Intelligence and Big-Data Convergence Center, Gil Medical Center, Gachon University College of Medicine, Incheon, South Korea; Instituto Nacional de Geriatria, MEXICO

## Abstract

**Purpose:**

We aimed to develop the Korean Hospital Frailty Risk Score (K-HFRS) by applying the International Classification of Diseases-10 codes to community-dwelling older adults’ medical data.

**Methods:**

We selected data from 2,761 people with no missing main variable values from the Korean Frailty and Aging Cohort Data (KFACD) and National Health Insurance Database (NHID) for analysis. Frailty was determined based on modified Fried’s phenotype [MFP] and Korean Frailty Index for Primary Care [KFI-PC] in the KFACD. A previously established method calculated the K-HFRS, verified by the area under the receiver operating characteristic (ROC) curve. The calculated cutoff value predicted the medical use.

**Results:**

The respective K-HFRSs of the frailty group using the MFP and KFI-PC criteria ranged from 3.64 (±3.03) to 8.15 (±5.72) and 4.07 (±3.42) to 9.10 (±6.28), with 7.67 (±5.40) and 8.59 (±6.03) when four diagnoses were included. The K-HFRS of the frailty group using the KFI-PC criteria was higher than that using the MFP criteria. With four diagnoses included using the MFP criteria, the adjusted odds ratio (OR) for medical expenditures in the frailty group compared to the non-frailty group was 3.01 (95% confidence interval [CI] 2.52–3.60, *p* < .001); for the number of emergency room (ER) visits was 2.19 (95% CI 1.77–2.70, *p* < .001); for inpatient days was 2.48 (95% CI 2.08–2.96, *p* < .001). With four diagnoses included using the KFI-PC criteria, the adjusted OR value for medical expenditures was 2.77 (95% CI 2.35–3.27, *p* < .001); for the number of ER visits was 1.87 (95% CI 1.51–2.32, *p* < .001); for inpatient days was 2.07 (95% CI 1.75–2.45, *p* < .001).

**Conclusion:**

This study substantiated that the K-HFRS can measure frailty efficiently at a lower cost. Follow-up studies are needed for additional validity.

## Introduction

As the average life expectancy has been extended due to improved living standards and the development of healthcare technology, the older population in South Korea has been substantially increasing, leading to rapid population aging in South Korea at a rate unprecedented in developed countries [1]. After entering an aging society in 2000, with 7.2% of the older population aged 65 y or older, South Korea joined an aged society in 2018 and is estimated to become a super-aged society by 2025 [2].

The increase in the older population leads to increased medical use and medical expenditures, which burden the national economy [3]. Thus, the health management project for the older population is presented as a national task. The South Korean government proposed the goals of community support and medical access enhancement for healthy old age in the 5th National Health Plan [4], and the World Health Organization (WHO) [5] announced a 10-y healthy aging project in 2021 [5].

Healthy aging refers to being able to engage in activities that people think are meaningful even when they are old, regardless of whether or not they have a disease [5]. This means that the conventional definition of health should be changed from disease-oriented to function-oriented and should emphasize the importance of maximally maintaining or improving physical function to delay the occurrence of frailty as much as possible [5].

Preventing functional decline in older individuals is necessary for healthy aging, and for this purpose, the prevention and management of frailty are crucial [5]. Frailty is a state in which the function of various organs and the reserve function that can recover is reduced, resulting in the increased physical functionality to the extent that it is impossible to live independently. Additionally, reduced physical functions do not recover appropriately, eventually leading to disease, requiring the care of others, and ultimately increasing the risk of death [6]. Considering the negative effects and health outcomes due to frailty, efforts to prevent and manage frailty are vital [7], and it is necessary to develop a frailty measurement tool that is valid and useful. Developing a low-cost, highly accessible frailty measurement tool can support primary medical institutions responsible for health projects for the older population in the community to detect and intervene in frailty at an early stage [8, 9]. This would also aid the South Korean government in its plan to provide frailty management services at these institutions [4]. General practitioners in the United Kingdom assess the level of frailty when treating older patients and manage frailty depending on the results to support the healthy life of people in old age [9].

As for the international frailty assessment tools, the modified version of Fried’s frailty phenotype proposed by Fried et al. [10] and the frailty index proposed by Rockwood et al. [11] are currently the most commonly introduced and used. However, the frailty phenotype proposed by Fried et al. [10], which consists of five criteria (weight loss, weakness, exhaustion, slowness, and low physical activity), requires additional personnel to measure grip strength and slowness (gait speed), and subjective interpretation is inevitable when interpreting low physical activity measurement results. Therefore, this manual frailty screening method has limited use in primary medical settings in South Korea [1, 12, 13]. The frailty index [11] by Rockwood et al., which consists of 70 items, is also somewhat difficult to use in primary medical settings due to the excessive number of items [1].

Regarding the frailty characteristics of older Korean individuals, South Korea developed and introduced the Korean Frailty Index (KFI) [1], the Korean Longitudinal Study on Health and Aging Frailty Index (KLoSHA) [14], and the Korean Frailty Index for Primary Care (KFI-PC) [15]. Among them, the KFI-PC [15] was developed for use in primary medical settings, including the assessment results of lack of nutrition, lack of physical exercise, sarcopenia, lack of social activity, and cognitive function decline. However, the KFI-PC also consists of 53 items, making it challenging to apply to primary medical settings. Since the KLoSHA [14] measures serum albumin levels and calculates the Korean Activity of Daily Living score and the Korean version of the Mini-Mental Status Examination score, applying it to the clinical field is difficult. The KFI [1] may be inappropriate to use alone because it is presented for screening before applying the KFI-PC [15]. Therefore, it is necessary to develop a frailty assessment tool that is convenient to use in primary medical institutions, takes less time, and can minimize measurement errors between people who take the measurements for primary medical institutions to be able to make early diagnoses and manage the frailty of all of the older populations they manage.

A previous study [8] developed and introduced the Hospital Frailty Risk Score, utilizing the International Statistical Classification of Disease and Related Health Problems, Tenth Revision (ICD-10) [16] generated in the treatment process. Studies [17–20] were performed to verify this. Since South Korea operates a single public insurer system for all its citizens, developing and utilizing the Hospital Frailty Risk Score using the ICD-10 can be much easier than in other countries. Thus, in this study, we aimed to examine whether the Korean Hospital Frailty Risk Score (K-HFRS) can be calculated using ICD-10 codes generated during the treatment process to seek methods to diagnose frailty effortlessly in the primary medical settings of the community.

## Materials and methods

### Study design

In this secondary data analysis study, we analyzed study data established by linking the Korean Frailty and Aging Cohort Data (KFACD) of the National Evidence-Based Healthcare Collaborating Agency (NECA) and the National Health Insurance Database (NHID).

### Data source

We used the data linking the NHID (2007–2019) of the National Health Insurance Service to the KFACD established by the Korean Frailty and Aging Cohort Study (KFACS). In the KFACS, 3,011 older individuals aged between 70 and 84 y residing in the community were enrolled in 2016 and 2017; the secondary investigation was performed after 2 y, respectively, in 2018 and 2019. The primary evaluation included frailty, health condition, health behavior, cognitive function, and social function. For the NHID involved in the analysis of this study, each individual’s data for a total of 2 y were assessed using the year the participant was enrolled in the cohort data (2016 or 2017) and the data for the following year after enrollment (2017 for those who enrolled in 2016; 2018 for those who enrolled in 2017). The participants included in the final analysis of this study were 2,761, who had no missing values in the main variables among the dataset generated in this way.

### Measurement

#### Korean Hospital Frailty Risk Score (K-HFRS)

To calculate the K-HFRS based on ICD-10 codes, 109 ICD-10 codes presented in the previous literature [8] and the points awarded for each code were used.

#### Frailty

Frailty status was assessed at the time of registration. Frailty was measured using the Korean Frailty Index for Primary Care (KFI-PC) [15] and the modified version of Fried’s frailty phenotype [10, 21], which was modified from the Cardiovascular Health Study and regularly tested by the Asia-Pacific region, including South Korea. The modified Fried’s phenotype consists of five criteria (weight loss, weakness, exhaustion, slowness, and low physical activity), and the study participants were classified as frail if they had three to five frailty criteria [10]. The KFI-PC is a Korean version of the frailty screening tool developed for use in primary medical settings in South Korea by referring to the Comprehensive Geriatric Assessment-based frailty index (FI-CGA) [22, 23], which was validated as suitable for assessing frailty in the primary medical field, and the validated Korean frailty indices [15]. It consists of a maximum of 53 deficit scores, with the score value from 0 to 1, and the tendency of frailty increases as the score increases [15]. This study classified a score of 0.23 or more (≥ 0.23) as frail [15].

#### Medical use

Medical use was calculated using the medical expenditure, number of emergency room (ER) visits, and inpatient days.

### Ethical considerations

This study was approved by the Institutional Review Board of the NECA (IRB No. NECA IRB21-020-8). This study complied with study data disclosure and utilization regulations of the NECA.

### Data analysis

The general characteristics, K-HFRS, and medical use (medical expenditures, number of ER visits, and inpatient days) of the participants and the frailty group determined via the modified Fried’s phenotype and KFI-PC criteria among them were presented as frequencies, percentages, means, and standard deviations using descriptive statistics.

According to the hospital frailty risk score presented by the previous study [8], the K-HFRS was calculated, and the following steps were followed to verify this. First, the area under the receiver operating characteristic (ROC) curve (AUC) value and the cutoff value with K-HFRS were calculated considering the frailty group based on two frailty criteria (modified Fried’s phenotype or KFI-PC) through the ROC analysis and the number of diagnoses included in one claim unit from a minimum of one (including only the principal diagnosis) to a maximum of five (including the principal diagnosis and four additional diagnoses). Second, to identify how accurately the cutoff values generated in the first step predict medical use, ROC analysis was performed with medical expenditure, number of ER visits, and inpatient days as dependent variables based on the cutoff values calculated in the first step to calculate the AUC and cutoff values. Afterward, they were converted to binary variables for Cohen`s Kappa logistic regression analysis. At that time, the effects of age, sex, and Charlson co-morbidity index (CCI) were adjusted. SAS EnterprizeGuide7.1 (SAS 9.3) was used to perform the analysis.

## Results

Of the 2,761 individuals in this study, the frailty group comprised 293 (10.61%) and 471 (17.1%) based on modified Fried’s phenotype and KFI-PC criteria, respectively. The mean age of the study population was 75.96 (±3.89) y, with the most common age group from 70–75 (48.9%) y. Females (51.8%) numbered more than males, and those who were unmarried (67.5%) were prevalent. Regarding the cohabitation status, living with only the spouse (51.8%) was the most common, followed by living alone (23.7%). Many had no religion (56.5%), and in terms of educational attainment, elementary or lower was the most common (46.4%). The highest household monthly income was less than 1 million won (43.7%), and regarding economic activity, most were employed (73.3%). The CCI reflecting the level of comorbidities was 1.79 (±1.51) (Table 1).

**Table 1:** Baseline characteristics.

	Total		Frailty group			
			By modified Fried’s phenotype		By KFI-PC	
	**N**	**(%)**	**N**	**(%)**	**N**	**(%)**
**Total**	2,761	(100.0)	293	(10.6)	471	(17.1)
**Age**	75.96±3.89		78.12±3.65		75.96±3.89	
70~75	1,351	(48.9)	77	(26.3)	139	(29.5)
76~80	964	(34.9)	122	(41.6)	186	(39.5)
81~84	446	(16.2)	94	(32.1)	146	(31.0)
**Gender**						
Male	1,332	(48.2)	117	(39.9)	115	(24.4)
Female	1,429	(51.8)	176	(60.1)	356	(75.6)
**Spouse**						
Partnered	897	(32.5)	118	(40.3)	272	(57.8)
Single	1,864	(67.5)	175	(59.7)	199	(42.3)
**Cohabitation**						
Alone	655	(23.7)	92	(31.4)	217	(46.1)
Only spouse	1,429	(51.8)	130	(44.4)	155	(32.9)
Only children	250	(9.1)	28	(9.6)	53	(11.3)
Spouse & children	375	(13.6)	37	(12.6)	57	(12.1)
Etc.	52	(1.9)	6	(2.0)	9	(1.9)
**Religion**						
Follow	1,200	(43.5)	129	(44.0)	233	(49.5)
Do not follow	1,561	(56.5)	164	(56.0)	238	(50.5)
**Educational level**						
≤ Elementary	1,282	(46.4)	184	(62.8)	366	(77.7)
Middle school	426	(15.4)	32	(10.9)	46	(9.8)
High School	553	(20.0)	50	(17.1)	42	(8.9)
≥ College	500	(18.1)	27	(9.2)	17	(3.6)
**Household income/month** ^*^						
≤ 100	1,206	(43.7)	172	(58.7)	325	(69.0)
100~200	693	(25.1)	61	(20.8)	103	(21.9)
200~300	377	(13.7)	34	(11.6)	22	(4.7)
300~	485	(17.6)	26	(8.9)	21	(4.5)
**Economic activity**						
In employment	2,023	(73.3)	220	(75.1)	363	(77.1)
Unemployed	738	(26.7)	73	(24.9)	108	(22.9)
**CCI**	1.79±1.51		2.02±1.47		2.09±1.49	
0	563	(20.4)	39	(13.3)	63	(13.4)
1	783	(28.4)	78	(26.6)	113	(24.0)
2	683	(24.7)	86	(29.4)	139	(29.5)
≥3	732	(26.5)	90	(30.7)	156	(33.1)

*Unit: 10,000 Korean won (KRW)

CCI = Charlson co-morbidity index.

The mean ages of the frailty group were 78.12 (±3.65) y, according to the modified Fried’s phenotype criteria, and 75.96 (±3.89) y, according to the KFI-PC criteria, with the most common age group from 76 to 80 y in both groups (41.6% and 39.5%, respectively). In both groups, females (60.1% and 75.6%, respectively) were more than males, and many had no religion (56.0% and 50.5%, respectively). In terms of educational attainment, elementary or lower was the most common (62.8% and 77.7%, respectively), and the highest household monthly income group had less than 1 million won (58.7% and 69.0%, respectively). Regarding economic activity, most were employed (75.1% and 77.1%, respectively). Those who were unmarried (59.7%) were more in the frailty group than in the non-frailty group, according to the modified Fried’s phenotype criteria, whereas many of the frailty group, according to the KFI-PC criteria, had a spouse (57.8%). In terms of cohabitation status, living with only the spouse (44.4%) was the most common in the frailty group, according to the modified Fried’s phenotype criteria, while living alone (46.1%) was the most common in the frailty group, according to the KFI-PC criteria. The CCI of the frailty group according to the modified Fried’s phenotype criteria was 2.02 (±1.47), and that of the frailty group according to the KFI-PC criteria was 2.09 (±1.49) (Table 1).

As the number of diagnoses included in the formula for calculating the K-HFRS increased, the K-HFRS itself increased. The K-HFRS of the study population ranged from 2.94 (±2.90) to 6.60 (±5.14), and it was 6.23 (±4.91) when including four diagnoses (including the principal diagnosis and three additional diagnoses). The K-HFRS of the frailty group, according to the modified Fried’s phenotype criteria, ranged from 3.64 (±3.03) to 8.15(±5.72), and it was 7.67 (±5.40) when including four diagnoses. The K-HFRS of the frailty group, according to the KFI-PC criteria, ranged from 4.07 (±3.42) to 9.10 (±6.28), and it was 8.59 (±6.03) when including four diagnoses. The K-HFRS of the frailty group according to the KFI-PC criteria was higher than that of the frailty group according to the modified Fried’s phenotype criteria. The medical expenditures of the study population were 834,349 (±959,560) Korean won (KRW), the number of ER visits was 0.31 (±0.92), and the inpatient days were 5.70 (±19.11). The frailty group according to the KFI-PC criteria had higher medical expenditures, number of ER visits, and inpatient days than did the frailty group according to the modified Fried’s phenotype criteria (Table 2).

**Table 2:** Korean hospital frailty risk score and healthcare utilization.

	Total	Frailty group	
		By modified Fried’s phenotype	By KFI-PC
**K-HFRS**			
Score1	2.94±2.90	3.64±3.03	4.07±3.42
Score 2	3.94±3.08	5.61±4.14	6.24±4.63
Score 3	5.63±4.54	6.92±4.92	7.74±5.53
Score 4	6.23±4.91	7.67±5.40	8.59±6.03
Score 5	6.60±5.14	8.15±5.72	9.10±6.28
**Medical expenditures** ^*^	834,349±959,560	1,021,330±1,092,576	1,103,656±1,260,265
**Number of ER visits**	0.31±0.92	0.39±0.94	0.45±1.09
**Inpatient days**	5.70±19.11	8.18±28.19	10.77±25.46

Score 1: Including only the main diagnosis

Score 2: Including the main diagnosis and the first sub-diagnosis

Score 3: Including the main diagnosis and the first and second sub-diagnoses

Score 4: Including the main diagnosis and the sub-diagnosis from the 1st to the 3rd

Score 5: Including the main diagnosis and the sub-diagnosis from the 1st to the 4th

*Unit: Korean won (KRW)

K-HFRS = Korean Hospital Frailty Risk Score; KFI-PC = Korean Frailty Index for Primary Care; ER = emergency room.

When the cutoff value and AUC were obtained by varying the frailty diagnosis criteria and the number of disease names included, the AUC was the highest at score 3 (including the principal diagnosis and two additional diagnoses, AUC = 0.590, cutoff = 7.70) and at score 5 (including the principal diagnosis and four additional diagnoses, AUC = 0.590, cutoff = 7.50) and the lowest at 2 (including the principal diagnosis and first additional diagnosis, AUC = 0.585, cutoff = 5.70) when the modified Fried’s phenotype criteria were applied. The AUC at score 4 was 0.589, and the cutoff was 7.60. When the KFI-PC frailty criteria were applied, it was the highest at score 5 (AUC = 0655, cutoff = 7.10) and the lowest at 1 (including only the principal diagnosis, AUC = 0.632, cutoff = 1.70). The AUC at score 4 was 0.652, and the cutoff was 5.10 (Table 3).

**Table 3:** Cutoff value and AUC by frailty diagnosis criteria and scores.

	Modified Fried’s phenotype ≥ 3		KFI-PC ≥ 0.23	
	Cutoff	AUC	Cutoff	AUC
Score 1	1.90	0.587 (0.553–0.621)	1.70	0.632 (0.605–0.659)
Score 2	5.70	0.585 (0.551–0.619)	3.40	0.636 (0.609–0.663)
Score 3	7.70	0.590 (0.554–0.624)	4.50	0.650 (0.623–0.676)
Score 4	7.60	0.589 (0.554–0.624)	5.10	0.652 (0.625–0.679)
Score 5	7.50	0.590 (0.555–0.626)	7.10	0.655 (0.629–0.682)

Score 1: Including only the main diagnosis; Score 2: Including the main diagnosis and the first sub-diagnosis; Score 3: Including the main diagnosis and the first and second sub-diagnoses; Score 4: Including the main diagnosis and the sub-diagnosis from the 1st to the 3^rd^; Score 5: Including the main diagnosis and the sub-diagnosis from the 1st to the 4^th^.

AUC = area under the ROC (Receiver Operating Characteristic) curve; KFI-PC = Korean Frailty Index for Primary Care; CI = confidence interval.

To identify how accurately the cutoff values calculated in Table 3 predict medical use, the cutoff value and AUC of medical use (medical expenditures, number of ER visits, and inpatient days) were calculated by applying the cutoff values calculated in Table 3. The AUCs of medical use at score 4 when applying the modified Fried’s phenotype frailty criteria were medical expenditures = 0.704, number of ER visits = 0.580, and inpatient days = 0.649. The AUCs of medical use at score 4 when applying the KFI-PC criteria were medical expenditures = 0.692, number of ER visits = 0.560, and inpatient days = 0.619 (Table 4).

**Table 4:** Cutoff value and AUC of healthcare utilization by frailty diagnosis criteria and scores.

	Cutoff	AUC (95% CI)	Non-frailty		Frailty	
			N	(%)	N	(%)
**Modified Fried’s phenotype ≥ 3**						
**Score 1: 1.90**			1,228	(100.0)	1,533	(100.0)
Medical expenditures*	510,894.77	0.651(0.630–0.671)	469	(38.2)	954	(62.2)
Number of ER visits	1.58	0.547(0.533–0.561)	39	(3.2)	131	(8.6)
Inpatient days	12.72	0.586(0.567–0.604)	307	(25.0)	605	(39.5)
**Score 2: 5.70**			1,912	(100.0)	849	(100.0)
Medical expenditures*	698,576.00	0.684(0.662–0.705)	513	(26.8)	513	(60.4)
Number of ER visits	1	0.570(0.553–0.587)	235	(12.3)	235	(27.7)
Inpatient days	1	0.623(0.603–0.645)	170	(8.9)	170	(20.0)
**Score 3: 7.70**			2,054	(100.0)	707	(100.0)
Medical expenditures*	906,435.28	0.687(0.664–0.710)	467	(22.7)	381	(53.9)
Number of ER visits	1	0.589(0.570–0.608)	290	(14.1)	220	(31.1)
Inpatient days	1	0.643(0.621–0.665)	669	(32.6)	408	(57.7)
**Score 4: 7.60**			1,887	(100.0)	874	(100.0)
Medical expenditures*	743,258.62	0.704(0.683–0.726)	528	(28.0)	526	(60.2)
Number of ER visits	1	0.580(0.563–0.598)	257	(13.6)	253	(29.0)
Inpatient days	1	0.649(0.628–0.669)	580	(30.7)	497	(56.9)
**Score 5: 7.50**			1,797	(100.0)	964	(100.0)
Medical expenditures*	743,274.69	0.703(0.680–0.723)	487	(27.1)	567	(58.8)
Number of ER visits	1	0.574(0.559–0.591)	243	(13.5)	267	(27.7)
Inpatient days	1	0.643(0.623–0.663)	544	(30.3)	533	(55.3)
**KFI-PC ≥ 0.23**						
**Score 1: 1.70**			1,136	(100.0)	1,625	(100.0)
Medical expenditures*	510,916.47	0.651(0.630–0.672)	522	(46.0)	992	(61.1)
Number of ER visits	1.58	0.547(0.532–0.560)	15	(1.3)	61	(3.8)
Inpatient days	12.72	0.586(0.568–0.605)	35	(3.1)	120	(7.4)
**Score 2: 3.40**			1,253	(100.0)	1,508	(100.0)
Medical expenditures*	475,278.63	0.675(0.655–0.695)	495	(39.5)	1,008	(66.8)
Number of ER visits	1.2	0.559(0.545–0.573)	154	(12.3)	356	(23.6)
Inpatient days	6.1	0.601(0.583–0.619)	158	(12.6)	401	(26.6)
**Score 3: 4.50**			1,307	(100.0)	1,454	(100.0)
Medical expenditures*	539,009.00	0.684(0.664–0.704)	456	(34.9)	918	(63.1)
Number of ER visits	1	0.559(0.545–0.573)	164	(12.6)	346	(23.8)
Inpatient days	1	0.614(0.596–0.619)	371	(28.4)	706	(48.6)
**Score 4: 5.10**			1,323	(100.0)	1,438	(100.0)
Medical expenditures*	538,979.76	0.692(0.673–0.712)	454	(34.3)	922	(64.1)
Number of ER visits	1	0.560(0.545–0.575)	165	(12.5)	345	(24.0)
Inpatient days	1	0.619(0.601–0.637)	370	(28.0)	706	(49.1)
**Score 5: 7.10**			1,704	(100.0)	1,057	(100.0)
Medical expenditures*	658,816.28	0.700(0.680–0.720)	521	(30.6)	653	(61.8)
Number of ER visits	1	0.508(0.553–0.584)	230	(13.5)	280	(26.5)
Inpatient days	1	0.638(0.620–0.659)	503	(29.5)	574	(54.3)

Score 1: Including only the main diagnosis; Score 2: Including the main diagnosis and the first sub-diagnosis; Score 3: Including the main diagnosis and the first and second sub-diagnoses; Score 4: Including the main diagnosis and the sub-diagnosis from the 1st to the 3^rd^; Score 5: Including the main diagnosis and the sub-diagnosis from the 1st to the 4^th^.

*Unit: Korean won(KRW).

AUC = area under the ROC(Receiver Operating Characteristic) curve; ER = emergency room; CI = confidence interval; ER = emergency room.

The correlation between frailty and medical use was identified using the cutoff value of medical use calculated in Table 4. At score 4 with the application of the modified Fried’s phenotype frailty criteria, the adjusted OR value for medical expenditures in the frailty group compared to the non-frailty group was 3.01 (95% CI 2.52–3.60, *p* < .001), indicating the range of adjusted OR for medical expenditures scores being 2.25 to 3.05. The adjusted OR value for the number of ER visits was 2.19 (95% CI 1.77–2.70, *p* < .001), showing the range of adjusted OR for the number of ER visit scores being 1.90 to 2.34. The adjusted OR value for inpatient days was 2.48 (95% CI 2.08–2.96, *p* < .001), indicating the range of adjusted OR for inpatient days scores being 1.66 to 2.48. At score 4 with the application of the KFI-PC frailty criteria, the adjusted OR for medical expenditures was 2.77 (95% CI 2.35–3.27, *p* < .001), with the range of adjusted OR for medical expenditures scores being 2.25 to 2.94. The adjusted OR value for the number of ER visits was 1.87 (95% CI 1.51–2.32, *p* < .001), with the range of adjusted OR for the number of ER visits scores being 1.84 to 2.06. The adjusted OR value for inpatient days was 2.07 (95% CI 1.75–2.45, *p* < .001), with the range of adjusted OR for inpatient days scores being 1.89 to 2.38 (Table 5).

**Table 5:** Association between K-HFRS and healthcare utilization.

	Crude OR	95% CI	Adjusted OR	95% CI
**Modified Fried’s phenotype ≥ 3**				
**Score 1**				
Medical expenditures^†^	2.67	0.28–3.11*	2.25	1.92–2.64*
Number of ER visits	2.85	1.98–4.11*	2.28	1.56–3.34*
Inpatient days	1.96	1.66–2.31*	1.66	1.40–1.98*
**Score 2**				
Medical expenditures^†^	3.29	2.78–3.89*	2.55	2.13–3.04*
Number of ER visits	2.28	1.87–2.78*	1.90	1.54–2.35*
Inpatient days	2.86	2.26–3.62*	2.24	1.74–2.88*
**Score 3**				
Medical expenditures^†^	3.97	3.32–4.76*	3.05	2.52–3.70*
Number of ER visits	2.75	2.25–3.36*	2.34	1.89–2.91*
Inpatient days	2.83	2.37–3.36*	2.33	1.93–2.80*
**Score 4**				
Medical expenditures^†^	3.89	3.29–4.61*	3.01	2.52–3.60*
Number of ER visits	2.58	2.12–3.15*	2.19	1.77–2.70*
Inpatient days	2.97	2.52–3.51*	2.48	2.08–2.96*
**Score 5**				
Medical expenditures^†^	3.84	3.26–4.53*	2.96	2.49–3.53*
Number of ER visits	2.45	2.02–2.98*	2.06	1.67–2.54*
Inpatient days	2.85	2.42–3.35*	2.32	2.00–2.82*
**KFI-PC ≥ 0.23**				
**Score 1**				
Medical expenditures^†^	2.25	2.27–3.1*	2.25	1.91–2.65*
Number of ER visits	2.91	1.65–5.15*	2.06	1.14–3.74*
Inpatient days	2.51	1.71–3.66*	1.89	1.27–2.81*
**Score 2**				
Medical expenditures^†^	3.09	2.64–3.61*	2.54	2.16–2.99*
Number of ER visits	1.79	1.79–2.71*	1.90	1.53–2.36*
Inpatient days	2.05	2.05–3.07*	2.01	1.63–2.49*
**Score 3**				
Medical expenditures^†^	3.20	2.74–3.74*	2.57	2.18–3.03*
Number of ER visits	2.18	1.78–2.67*	1.84	1.48–2.29*
Inpatient days	2.38	2.03–2.79*	1.99	1.68–2.36*
**Score 4**				
Medical expenditures^†^	3.42	2.92–4.00*	2.77	2.35–3.27*
Number of ER visits	2.22	1.81–2.71*	1.87	1.51–2.32*
Inpatient days	2.47	2.11–2.90*	2.07	1.75–2.45*
**Score 5**				
Medical expenditures^†^	3.67	3.12–4.31*	2.94	2.48–3.49*
Number of ER visits	2.31	1.90–2.81*	1.94	1.57–2.39*
Inpatient days	2.84	2.42–3.33*	2.38	2.01–2.82*

*p < .001

Adjusted for Age, gender, Charlson co-morbidity index (CCI).

Score 1: Including only the main diagnosis; Score 2: Including the main diagnosis and the first sub-diagnosis; Score 3: Including the main diagnosis and the first and second sub-diagnoses; Score 4: Including the main diagnosis and the sub-diagnosis from the 1st to the 3^rd^; Score 5: Including the main diagnosis and the sub-diagnosis from the 1st to the 4^th^.

^†^Unit: Korean won (KRW).

AUC = area under the ROC(Receiver Operating Characteristic) curve; ER = emergency room; OR = odds ratio; CI = confidence interval: ER = emergency room.

## Discussion

We utilized ICD-10 codes to calculate the K-HFRS. Since South Korea utilizes a single national health insurance system and all medical institutions, including primary institutions, use a single computerized system, there is a special advantage in using the K-HFRS for frailty identification. In addition, identifying the frailty risk using ICD-10 codes has the advantage of reducing the burden on medical institutions as it does not require efforts to create additional manual scores by using essential data from patients generated by hospitals [8, 24].

We calculated the K-HFRS by varying the number of diagnoses included in the claims unit. The validity of the calculated values was calculated by comparing it with the frailty group diagnosed using two frailty diagnosis tools (modified Fried’s phenotype and KFI-PC). We determined that it was most appropriate to include four diagnoses (the principal diagnosis and up to three additional diagnoses) for K-HFRS calculation after many discussions. When the four diagnoses were included, the AUC value when diagnosed with the K-HFRS and modified Fried’s phenotype was 0.589, and the AUC value with the KFI-PC was 0.652. A previous study conducted in the United Kingdom [8] included only the first diagnosis from the inpatient data to calculate the hospital frailty risk score, indicating the kappa score at 0.22 with Fried’s phenotype and 0.30 with the Rockwood classification. Although all AUC values presented in this study were below 0.70, which was not high, it can claim to present improved values compared to the previous study conducted in the United Kingdom [8]. This finding may be related to the fact that we used the ICD-10 codes generated from all the participants’ medical data for 2 y, whereas the previous study [8] only used the inpatient data [25]. At the time of writing, South Korea is attempting to calculate the K-HFRS for the first-time using ICD-10 codes. Follow-up studies should verify the validity of the K-HFRS developed in this study and develop the new methodology simultaneously.

In this study, the AUC value for medical expenditures was 0.704 when the modified Fried’s phenotype frailty criteria were applied and up to four diagnoses were included, and that for medical expenditures was 0.692 when the KFI-PC frailty criteria were applied and up to four diagnoses were included. These results are impossible to compare directly because there are no previous studies, but it can be said that the AUC value of 0.69 or more in both results is acceptable. However, further studies are required to seek methods to improve this. Moreover, because of this study, the AUC values for the number of ER visits and inpatient days were both 1 when applying the modified Fried’s phenotype and the KFI-PC criteria. This finding is assumed to be because most patients visited the ER once, and their number was small (M±SD of the number of ER visits = 0.31±0.92); inpatient days value was 1 because not many participants experienced hospitalization. Thus, follow-up studies with enough participants are needed.

Frailty is associated with negative health outcomes, such as hospitalization [26], increased medical expenditures [27], severe functional impairment [18], and decreased quality of life [18]. When applying the modified Fried’s phenotype and the KFI-PC frailty criteria in this study, the adjusted OR value for medical expenditures in the frailty group compared to the non-frailty group ranged from 2.25 to 3.01, that for number of ER visits ranged from 1.84 to 2.28, and that for inpatient days ranged from 1.66 to 2.48. These study results are consistent with the results of previous studies [8, 18, 26, 27], suggesting that frailty is related to increased use of medical resources. Medical resources should be managed by actively preventing and managing frailty, and it would be most effective for such an attempt to be implemented in the primary medical field with the highest access to medical use.

We calculated the K-HFRS by applying the modified Fried’s phenotype and KFI-PC frailty criteria. Both the K-HFRS and AUC value were higher when applying the KFI-PC frailty criteria than the modified Fried’s phenotype criteria. The modified Fried’s phenotype frailty criteria are commonly used worldwide to define frailty. In addition, they are used as a comparison index in studies developing frailty scales and aid in verifying their validity [1, 8, 15, 28]. On the other hand, the KFI-PC was developed in South Korea to identify frailty in older individuals in the community at primary medical institutions [15]. However, limitations should be considered since the validity is ensured, yet it is not highly utilized. In addition, in the study that developed the KFI-PC [15], the modified Fried’s phenotype frailty criteria were used to verify the validity. This fact should be considered in interpreting the study results and follow-up studies.

To date, frailty has not been routinely assessed in older individuals in the South Korean medical field. South Korea, which is expected to enter a super-aged society in 2025, needs frailty screening for older individuals at the community level to establish a resource allocation plan at the national level and identify patients most in need of benefits from the older people care projects. To this end, developing the frailty screening method using ICD-10 codes generated during the treatment process is valuable as frailty can be detected earlier at a low cost without additional effort. Hence, this study developed the K-HFRS based on ICD-10 codes. However, this study had limitations as follows. First, since the 109 ICD-10 codes used in this study for diagnosing frailty were developed in the United Kingdom, there was a limitation in applying them to South Korean older people. Yet, in South Korea, where the use of ICD-10 codes is not considered at all for diagnosing frailty, this study suggested the possibility of using ICD-10 codes for diagnosing frailty. Further discussion is required to prepare a Korean frailty diagnosis ICD-10 code set. Second, we developed the K-HFRS only for those registered in the KFACD. Hence, the results of this study cannot be generalized to all older South Korean individuals. It is necessary to identify the results of this study by utilizing representative older people data in South Korea. Third, calculating the frailty risk score based on ICD-10 codes in those with relatively limited medical use can raise a potential disadvantage, while the frailty risk score can be estimated as relatively low [8]. However, considering that the burden of visiting a medical institution is less in South Korea due to the application of national health insurance, the South Korean government is currently implementing various projects to improve access to healthcare among older people to enhance their quality of life [4]. Additionally, as of 2021, 43.4% of total medical expenditures are spent by older individuals aged 65 y or older, and it can be regarded that the barriers to using medical institutions for older individuals are relatively low compared to other countries [29]. Thus, calculating the frailty risk score based on ICD-10 codes may have higher validity in South Korea than in other countries. Fourth, the HFRS cannot consider the severity of the patient’s current condition [30]. A previous study [20] has reported the limited value of the HFRS for risk prediction in patients in the intensive care unit whose outcome is determined by the severity of the acute condition. We propose to use the K-HFRS as a primary frailty screening tool in primary medical settings considering these limitations of the HFRS. Lastly, ICD-10 codes of patients generated during the treatment process were not data generated for study purposes. Therefore, it should be considered that there may be errors in entering the diagnosis.

In conclusion, we developed the K-HFRS based on ICD-10 codes, identified the correlation between the modified Fried’s phenotype, which is most widely distributed internationally for frailty measurement, and the KFI-PC, which was developed for use in primary medical institutions in South Korea and suggested the association with medical use. The results of this study presented evidence that the K-HFRS can more easily measure frailty in community-based primary medical institutions.
